# Kinetic variables of the lower limb joint that affect the drop jump index at different drop heights

**DOI:** 10.3389/fspor.2024.1458722

**Published:** 2024-11-19

**Authors:** Takuya Yoshida, Amane Zushi, Kodayu Zushi, Ryohei Hayashi, Hirohiko Maemura, Satoru Tanigawa

**Affiliations:** ^1^ Department of Sport Science and Research, Japan Institute of Sport Sciences, Tokyo, Japan; ^2^ Faculty of Economics, Shiga University, Shiga, Japan; ^3^ Faculty of Education, Gifu University, Gifu, Japan; ^4^ Faculty of Health and Sport Sciences, University of Tsukuba, Ibaraki, Japan

**Keywords:** plyometrics, stretch-shortening cycle, joint mechanics, elite athlete, jump event, rebound jump

## Abstract

**Background:**

The drop jump index evaluates power exertion in the lower limb stretch-shortening cycle. In addition, the ability to exert power during the stretch-shortening cycle can be evaluated in detail by combining the drop jump index with the kinetic variables of the three lower limb joints. The purpose of this study was to determine the kinetic variables of the three lower limb joints during takeoff that affect the drop jump index of a drop jump from different drop heights.

**Methods:**

In total, 100 male athletes performed drop jumps from three drop heights (0.3, 0.6, and 0.9 m). Drop jump index and kinetic variables (torque, power, and work) of the three lower limb joints were calculated using body coordinates by infrared camera, and ground reaction force data by force plate. Multiple regression analysis was used to examine the parameters by which the kinetic variables of the three lower limb joints affected the drop jump index.

**Results:**

As a result, ankle joint and knee joint positive power were extracted as parameters affecting drop jump index at 0.3 m. In addition to these parameters, ankle negative power, ankle negative work and hip eccentric torque at 0.6 m, and knee eccentric torque at 0.9 m were extracted as parameters affecting the drop jump index.

**Conclusions:**

These results suggest that a higher drop height leads to a greater effect of eccentric torque exertion at the knee and hip joints and of positive power at the ankle and knee joints on the acquisition of the drop jump index.

## 1 Introduction

The ability to exert power in the stretch-shortening cycle (SSC) of the lower limbs affects basic motor performance, including sprinting, change of direction, and jumping in various sports (1–6). This ability is assessed using indicators such as the drop jump (DJ) index (7, 8) and rebound jump (RJ) index (9, 10) of DJ or RJ. Individuals with high athletic abilities for sprinting, change of direction, and jumping have a higher RJ-index or DJ-index (8, 9, 11, 12). Therefore, the evaluation and improvement of the ability to exert power in lower limb SSC is important for athletes in various sports (2, 10, 13). Additionally, the DJ-index and RJ-index comprehensively evaluate the ability to exert power in lower limb SSC of the three lower limb joints (ankle, knee, and hip) (10, 14, 15). DJ and RJ with jump heights as high as possible in the shortest duration exert great power at the ankle and knee joints (10, 16–19). In particular, the joint power exerted by the ankle joint during the concentric phase of an RJ considerably affects the RJ-index, contact time, and jump height (10). In addition, variables mainly related to the ankle joint affects contact time, whereas variables mainly related to the hip and knee joints affect the jump height (10). On the other hand, DJ can pre-control the stretch load at ground contact with the drop height; the kinetic variables of the three lower limb joints that affect the DJ-index are speculated to be different from RJ, where the own jump height is the stretch load. Therefore, clarifying the kinetic variables of the three lower limb joints during takeoff in DJ at different drop heights will provide insight into the detailed assessment of the ability to exert power in lower limb SSC using DJ and the consequent prescription of effective plyometric training.

The DJ is subjected to an increased stretch load at ground contact with an increase in the drop height. As the DJ drop height increases, the performance variables, including DJ-index, and kinetic variables of the three lower limb joints change. For example, if the drop height is increased to a level that exceeds the optimal height for the participant, the contact time increase, and the DJ-index and jump height also decrease (8, 17, 20–26). For the kinetic variables of the three lower limb joints, with the increase in contact time due to the increase in drop height, the ankle and knee joint flexions increase in the eccentric phase (27), and the torque and negative power and work of the three lower limb joints increase in the eccentric phase (17, 23). Moreover, the torque and power exerted by the ankle joint decrease in the concentric phase (17). This decrease is due to the increase in stretch load and the resulting increase in eccentric extension torque and power as the drop height increases. When the stretch load becomes excessive, unable to resist the stretch load, the lower limb joints flex greatly to absorb the impact, and the contact time also increases (20, 23, 26). In addition, the mechanisms that exert power via SSC, such as the stretch reflex and elastic energy, do not work effectively (25, 28–30), and the torque and power generated by the increase in eccentric extension torque and power due to the increase in stretch load are not transmitted to the concentric phase. In contrast, athletes (top level jump event athletes who compete in international competitions, etc) with superior ability to exert power in lower limb SSC have the highest jump heights in DJ from a higher drop height (higher than their own jump height) (8). Furthermore, the lack of decrease in the DJ-index at low to high drop heights is related to athletic performance in jump events. Therefore, the acquisition of a high DJ-index at a high drop height may exhibit characteristics different from previously reported changes in kinetic variables of the three lower limb joints from low to high drop heights. In order to achieve a short contact time in DJ, it is necessary to minimize the flexion of the lower limb joints during the takeoff phase (19, 31). Therefore, to achieve a higher DJ index at a higher drop height, it is speculated that torque and power must be generated to minimize knee, hip, and ankle flexion during the takeoff phase. Furthermore, previous studies have reported that in athletes with high DJ performance, the state of intracortical inhibition that controls the ankle joint muscles during the pre-set phase becomes a state of disinhibition, and this becomes more pronounced as the height of the takeoff increases (32). It has also been suggested that the state of disinhibition during the pre-set phase affects the facilitation of the stretch reflex during the takeoff phase and the large torque exerted by the ankle joint muscles during the same phase (33). Therefore, the elite athletes with superior power exertion ability in the lower limb SSC may be able to exert greater power even under greater stretch loads by effectively activating the force transmission mechanism via SSC, such as the stretch reflex during takeoff. Thus, in addition to average data from a large number of athletes, it would be useful to present characteristics of DJ performance and kinetic variables of the three lower limb joints in elite athletes with superior ability to exert power in the lower limb SSC. This is an important perspective when assessing the ability of high-performance athletes to power exertion during the lower limb SSC.

The present study aimed to determine the lower limb joint kinetics that affect the DJ-index in DJ at different drop heights. We hypothesized that eccentric torque and positive power exertion affect the acquisition of a higher DJ-index with increasing drop height. At higher drop heights, the torque exerted at not only the ankle and knee joints, but also the hip joint may influence the acquisition of the DJ-index.

## 2 Materials and methods

### 2.1 Ethical approval

This study was approved by the Ethics Committee of the Faculty of Health and Sports Sciences, University of Tsukuba (approval number: tai30-142, date of approval: April 25, 2019).

Before starting the experiment, all participants were fully informed about the purpose, methods, and possible risks of participating in the experiment of the study prior to signing an institutionally approved informed consent document. The participants were informed of the test and allowed to sufficiently practice the DJ in advance.

### 2.2 Participants

One-hundred male athletes (age, 20.6 ± 1.4 years; height, 1.77 ± 0.08 m; weight, 72.7 ± 10.3 kg) were enrolled. All participants were members of university sports clubs or club teams (badminton: 14 (1), basketball (1): 18, handball: 15(1), volleyball: 12(1), jump event: 4(4), tennis: 16(1), soccer: 21(4)). The number in brackets indicates the number of players who have competed in international competitions. We included athletes who had competed in their respective sports from the first division of university into international competitions (including the Olympic games). These participants trained approximately 5 days a week at their clubs. In this study, we present individual data of two participants who showed characteristic trends in the DJ-index results described below: participant A (Sub. A) was a top athlete who participated in international track and field jump events competitions; participant B (Sub. B) was a badminton student athlete. All participants voluntarily enrolled in the study, and thus, monetary compensation was not provided. The exclusion criteria were the use of medications affecting exercise capacity, orthopedic limitations, and a history of a major injury. The participants were familiar with the experimental procedures and had experience with strength and plyometric training, including DJ. The participants followed a normal training program during the study and did not engage in any strenuous exercise the day before the measurements were performed.

### 2.3 Experimental protocol

**Figure 1:**
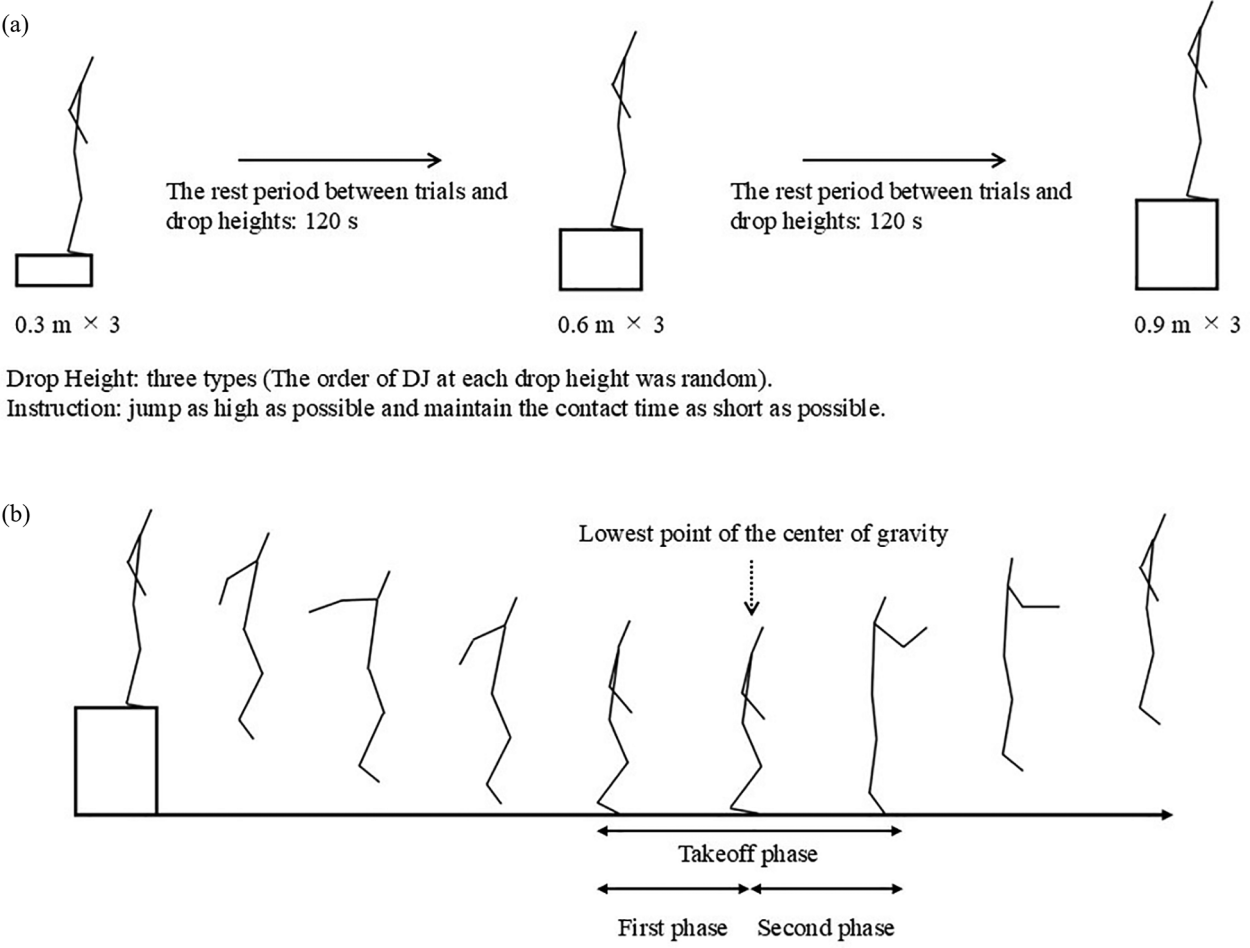
Drop jump experimental diagram.

All measurements were performed in the muscle strength and power measurement room. All participants were instructed to refrain from resistance training the day before the experiment. Before the experiment, they had to perform 15 min of low-intensity jogging, including dynamic stretching. Before the experiment, the participants practiced to ensure that they could perform the trials correctly, according to the instructions of an author who was familiar with the experimental protocol. The athletes twice practiced DJ in advance to minimize the changes in performance due to the learning effect occurring during measurement and to suppress variations in jumping techniques (34). The participants then practiced DJ twice before the actual performance of each test from three drop heights (0.3, 0.6, and 0.9 m). Three DJ trials for each drop height were then performed with 120 s of rest between trials (Figure 1a). The order of DJ at each drop height was random. The participants were instructed to place their feet separately on two force plates (Kistler 9287C; Kistler Instrumente AG, Winterthur, Switzerland). The participants were instructed to jump as high as possible and maintain the contact time as short as possible. Considering those participants in this study typically plyometric training using arm swings, in all the trials, free swinging was used without any restrictions on the swinging motion of the arms to avoid variability in jumping technique. The jump with the highest DJ-index at each drop height was chosen for statistical analysis. A failed DJ attempt was defined as touching the ground outside the force plate or contact time exceeds 0.250 s, and this contact time is defined as the threshold for fast SSC (35); in such cases, additional trials were conducted. To perform measurements on the two force plates when stepping on each with the left and right feet, the measurer visually checked the task, and a video was recorded to confirm that the stepping was performed correctly. The rest period between trials was 120 s (2 min) to account for the effects of fatigue (36) and the immediate effects of the last trial. Participants were not provided any feedback regarding their performance.

### 2.4 Data analysis

The three-dimensional coordinates of 13 retro-reflective markers (diameter: 14 mm) fixed on the body were collected using a Vicon T20 system (Nexus 2; Vicon Motion Systems, Ltd., Oxford, UK) with 10 cameras operating at 250 Hz. Auto-labeling was used for the measurement of body coordinates. In the preliminary experiment, due to the expectation that the coordinates of the left and right hip joint markers would be swapped during the measurement, a dummy marker was attached to one thigh to avoid the problem. If the coordinates of the hip joint markers were swapped during the trial, the swapped coordinates were re-labeled. The reflective marker was fixed at the periphery with kinesiology tape (NITREAT Kinesiology Tape; Nitto Group Company, Osaka, Japan) to ensure that the marker would not be removed during the measurement.

Ground reaction force (GRF) was measured using two force platforms (Kistler 9287C, 0.9 m × 0.6 m; Kistler Instrumente AG) at 1,000 Hz. These data were time-synchronized using Vicon Nexus software (Nexus 2; Vicon Motion Systems, Ltd.) for the subsequent inverse dynamic analysis. The kinetic measurements of the dominant leg were used for data analysis.

The ground contact and air times were calculated at the point where the vertical GRF was <10 N. The jump height was calculated using the following free-fall formula: jump height = (g · tair^2^) 8^−1^, with “g” as the gravitational acceleration with a value of 9.81 m/s^2^. The DJ-index was calculated by dividing the jump height by the contact time (9, 14, 15).

Twelve representative body points (10) were used for each participant (1: right toe, 2: right heel, 3: right ankle, 4: right knee, 5: right greater trochanter, 6: right shoulder, 7: left toe, 8: left heel, 9: left ankle, 10: left knee, 11: left greater trochanter, and 12: left shoulder), and one dummy marker was attached to the left thigh. The global coordinate system was defined using the participants’ jumping directions in the X (mediolateral direction), Y (anterior–posterior direction), and Z (vertical direction) axes. We used the same coordinate system as Zushi et al. (10) to calculate the joint torque and joint angle.

The coordinates were smoothened using a fourth-order, zero-lag, low-pass Butterworth filter with optimal cut-off frequencies of 7.5–15 Hz, which were determined using the residual method (37). The center of mass and inertial parameters were estimated on the basis of the body-segment parameters of Japanese athletes (38). The ankle joints were analyzed for plantarflexion and dorsiflexion, and the knee and hip joints were analyzed for extension and flexion. The ankle joint angle was defined as the angle between the line segment connecting the ankle and knee and that connecting the ankle and toe. The knee joint angle was defined as the angle between the line segment connecting the knee and greater trochanter and that connecting the knee and ankle. The hip joint angle was defined as the angle formed by the line segment connecting the greater trochanter and shoulder and that connecting the greater trochanter and knee. Angular velocity was calculated as the average of negative and positive values for each joint, setting the flexion velocity as the negative value and extension velocity as the positive value.

Joint kinetics was divided into the first and second halves of the takeoff based on the lowest point of the center of gravity (Figure 1b). Joint torque was calculated using an inverse dynamics approach. The torque at each joint was transformed into the joint coordinate system (10). The joint power was computed as the dot product of the joint torque and joint angular velocity, and the average values of the negative and positive powers due to the extension torque during the takeoff phase were calculated. The negative and positive joint work values were calculated by integrating the power over time. These data were determined at the positive extension–negative flexion (positive plantarflexion–negative dorsiflexion) axis around the ankle, knee, and hip joints (10, 14, 15).

### 2.5 Statistical analysis

Intraclass correlation coefficients (ICCs) were calculated to determine the inter-measurement reliability of the measured variables. Furthermore, the mean error (ME), the mean absolute error (MAE), and the mean absolute percentage error (MAPE) between measurements were calculated. Data were normally distributed for all conditions, assessed by the Kolmogorov-Smirnov test. Pearson's product rate correlation coefficient was used to analyze the correlation between the DJ-index and jump height and contact time. By referring to a previous study (10), multiple regression analysis was performed to examine the effects of the kinetic variables of the three lower limb joints on the DJ-index, with the DJ-index as the dependent variable and kinetic variables of the three lower limb joints as the independent variables. For the determination of these parameters, we referred to the kinetic variables of the lower limb joints that were used in previous studies (10, 14–17, 19). The alpha level was set at 0.05. All data are presented as mean ± standard deviation (SD). Statistical analyses were performed using SPSS (version 29, IBM Corp., NY, USA).

## 3 Results

The inter-measurement reliability of the DJ-index in this study was high (ICC: 0.3 m: 0.972 (0.959–0.981, 95% CI), 0.6 m: 0.986 (0.979–0.991, 95% CI), and 0.9 m: 0.954 (0.932–0.968, 95% CI)). The ME, MAE and MAPE of the DJ-index for each drop height were investigated, with the following results: a ME = 0.010 m/s, a MAE = 0.111 m/s and a MAPE = 5.387% at 0.3 m, a ME = 0.009 m/s, a MAE = 0.104 m/s and a MAPE = 4.922% at 0.6 m, a ME = 0.017 m/s, a MAE = 0.138 m/s and a MAPE = 6.882% at 0.9 m. The test of normality values of the DJ-index were 0.188, 0.344, and 0.212 for drop heights of 0.3, 0.6, and 0.9 m, respectively.

Tables 1, 2 shows the calculated variables as mean ± SD. The mean data showed that the DJ-index and jump height were highest at 0.6 m, and the contact time was shortest at 0.3 m. The results for Sub. A and B show that the DJ-index and jump height of Sub. A tended to increase as the drop height increased, at 0.9 m, the DJ-index was highest at 0.9 m among all participants. In contrast, Sub. B showed the highest DJ-index of all participants at 0.3 m, but the DJ-index and jump height tended to decrease as the drop height increased.

**Table 1:** Drop jump parameters at each drop height.

		0.3 m								0.6 m								0.9 m							
Parameters		Mean	SD	95% CI		Max	Min	Sub.A	Sub.B	Mean	SD	95% CI		Max	Min	Sub.A	Sub.B	Mean	SD	95% CI		Max	Min	Sub.A	Sub.B
Performance variables																									
DJ-index		2.13	0.44	2.04	2.22	3.60	1.12	2.96	3.60	2.16	0.54	2.04	2.26	3.63	1.02	3.30	3.48	1.94	0.56	1.82	2.05	3.70	0.83	3.70	2.60
Jump height (m)		0.42	0.06	0.40	0.43	0.53	0.28	0.51	0.49	0.42	0.07	0.41	0.44	0.59	0.26	0.58	0.46	0.40	0.08	0.39	0.42	0.61	0.20	0.61	0.45
Contact time (s)		0.199	0.025	0.193	0.203	0.250	0.136	0.172	0.136	0.204	0.028	0.206	0.222	0.248	0.132	0.176	0.132	0.214	0.004	0.206	0.222	0.250	0.148	0.164	0.172
Eccentric torque (Nm/kg)																									
Hip		1.51	0.81	1.25	1.65	3.64	0.12	1.22	0.29	1.88	0.84	1.65	2.05	5.07	0.32	1.92	3.25	2.40	0.98	2.21	2.72	6.46	0.73	3.43	4.06
Knee		2.06	0.46	2.01	2.24	3.65	1.34	1.72	3.35	2.58	0.55	2.55	2.81	4.32	1.62	3.51	4.11	3.11	0.71	3.08	3.42	5.83	1.29	4.18	4.07
Ankle		2.21	0.42	2.22	2.41	3.26	1.21	1.21	2.96	2.65	0.60	2.66	2.94	5.45	1.75	3.32	4.15	2.96	1.19	2.86	3.48	11.34	0.97	4.41	3.74
Concentric torque (Nm/kg)																									
Hip		1.07	0.60	0.96	1.25	3.56	0.17	0.66	1.44	1.05	0.44	0.97	1.19	3.40	0.41	1.33	1.42	1.05	0.45	0.94	1.16	2.55	0.17	1.42	1.03
Knee		2.10	0.42	2.04	2.22	3.71	1.49	1.72	2.60	2.24	0.50	2.20	2.42	3.66	1.32	3.19	2.80	2.28	0.69	2.04	2.22	3.71	1.49	3.33	2.64
Ankle		2.08	0.37	2.08	2.25	2.90	1.16	1.16	2.45	2.20	0.41	2.20	2.39	3.97	1.53	2.67	2.16	2.14	0.59	2.06	2.37	6.19	0.96	2.64	1.79
Negative power (W/kg)																									
Hip		−2.21	2.06	−2.32	−1.38	−8.00	4.83	−4.34	0.53	−5.41	5.23	−5.60	−3.67	−24.45	0.42	−0.16	−6.16	−10.59	6.60	−12.39	−8.96	−37.76	3.28	−5.67	−32.61
Knee		−8.99	3.57	−9.26	−7.88	−16.95	−3.89	−7.68	−8.46	−15.68	4.94	−16.94	−14.70	−30.63	−8.13	−13.71	−25.38	−24.79	7.68	−27.97	−24.45	−44.81	−9.33	−21.69	−44.43
Ankle		−12.83	5.18	−15.53	−13.06	−27.38	−5.04	−5.04	−21.72	−17.70	6.86	−21.35	−18.12	−41.00	−7.19	−23.44	−24.46	−21.09	10.48	−26.15	−20.88	−71.25	−2.96	−33.06	−27.33
Positive power (W/kg)																									
Hip		3.43	1.81	3.14	3.97	8.08	0.69	1.51	5.65	3.55	1.71	3.24	4.10	9.86	0.54	2.97	7.14	3.51	1.79	3.07	3.95	10.89	0.08	6.80	4.63
Knee		12.07	3.16	11.90	13.33	26.41	6.22	9.53	18.30	12.92	3.89	12.97	14.76	26.54	5.60	17.75	17.63	11.95	5.19	11.71	14.30	42.69	3.54	22.10	17.53
Ankle		11.05	2.86	11.14	12.49	17.99	6.37	6.88	17.99	11.83	3.61	11.81	13.50	25.53	5.74	13.49	18.18	11.28	4.35	10.99	13.15	36.26	3.55	15.37	9.92
Negative work (J/kg)																									
Hip		−0.12	0.12	−0.11	−0.07	0.00	−0.34	−0.34	−0.02	−0.27	0.26	−0.25	−0.17	−0.01	−0.72	−0.03	−0.10	−0.54	0.41	1.25	1.36	1.86	0.85	−0.18	−0.79
Knee		−0.69	0.29	−0.68	−0.58	−0.13	−1.36	−0.75	−0.45	−1.20	0.33	−1.24	−1.09	−0.47	−2.08	−0.89	−1.12	−1.88	0.42	−1.97	−1.79	−1.23	−2.82	−1.37	−2.82
Ankle		−0.91	0.22	−0.99	−0.88	−0.51	−1.63	−0.62	−0.90	−1.30	0.30	−1.41	−1.27	−0.88	−2.21	−1.29	−1.00	−1.68	0.53	−1.87	−1.60	−0.76	−4.47	−1.53	−1.13
Positive work (J/kg)																									
Hip		0.40	0.28	0.32	0.45	1.23	0.03	0.12	0.39	0.39	0.27	0.30	0.43	1.62	0.02	0.29	0.31	0.42	0.30	0.31	0.45	1.84	0.00	0.48	0.31
Knee		1.09	0.31	1.02	1.13	1.79	0.57	1.07	1.09	1.15	0.25	1.11	1.23	1.76	0.63	1.42	1.06	1.17	0.33	1.13	1.28	2.74	0.65	1.51	1.33
Ankle		1.25	0.23	1.25	1.36	1.86	0.85	0.85	1.51	1.35	0.26	1.34	1.46	2.15	0.92	1.62	1.39	1.36	0.36	1.30	1.49	3.77	0.71	1.74	1.25

**Table 2:** Joint flexion and extension parameters during takeoff at each drop height.

	0.3 m								0.6 m								0.9 m							
Parameters	Mean	SD	95% CI		Max	Min	Sub.A	Sub.B	Mean	SD	95% CI		Max	Min	Sub.A	Sub.B	Mean	SD	95% CI		Max	Min	Sub.A	Sub.B
Joint Flexion (deg)																								
Hip	2.66	4.16	1.84	3.48	17.40	0.00	0.00	0.00	5.85	7.06	4.46	7.24	36.90	0.20	0.20	1.80	12.60	10.13	10.61	14.59	56.20	0.20	0.20	10.00
Knee	24.65	8.29	23.02	26.28	43.30	5.00	11.30	9.30	31.92	8.99	30.15	33.68	63.90	13.00	18.80	15.20	39.67	10.01	37.71	41.64	79.10	9.30	18.00	40.10
Ankle	34.88	5.78	33.75	36.02	50.90	21.60	21.60	31.30	43.43	6.97	42.06	44.80	60.70	24.00	29.80	37.40	49.55	7.00	48.18	50.93	65.30	10.70	36.70	48.70
Joint extension (deg)																								
Hip	36.88	11.57	34.60	39.15	65.70	7.70	46.00	39.40	34.52	11.19	32.32	36.72	61.30	3.80	39.20	23.60	35.36	14.91	32.43	38.29	84.80	12.60	34.50	39.00
Knee	55.54	8.66	53.84	57.24	74.90	24.40	56.90	49.70	58.12	8.38	56.47	59.76	81.70	32.10	57.20	44.60	61.12	9.07	59.34	62.90	94.60	33.90	57.20	62.00
Ankle	54.42	7.79	52.89	55.95	70.10	0.00	51.20	61.10	56.56	5.24	55.53	57.59	71.50	40.40	54.10	58.40	57.53	6.48	56.26	58.81	71.70	15.50	53.60	64.80

Table 3 shows the correlation between the DJ-index, jump height, and contact time at each drop height. In 0.3 m, a significantly high correlation was found between the DJ-index and jumping height (r = 0.769, *P* < 0.001) and between the DJ-index and contact time (r = −0.688, *P* < 0.001), while no correlation was found between the jump height and contact time (r = −0.092, *P* = 0.360, n.s.). In 0.6 m, a significantly high correlation was found between the DJ-index and jump height (r = 0.826, *P* < 0.001) and between the DJ-index and contact time (r = −0.788, *P* < 0.001), while a small correlation was found between the jump height and contact time (r = −0.341, *P* < 0.001). In 0.9 m, a significantly high correlation was found between the DJ-index and jump height (r = 0.852, *P* < 0.001) and between the DJ-index and contact time (r = −0.668, *P* < 0.001), while a small correlation was found between the jump height and contact time (r = −0.246, *P* < 0.014).

**Table 3:** The correlations among the drop jump performance variables at each drop height.

0.3 m				
	Jump height		Contact time	
	r	*p*-value	r	*p*-value
DJ-index	0.769**	0.001	−0.688**	0.001
Jump height (m)			−0.092	0.360
0.6 m				
	r	*p*-value	r	*P*-value
DJ-index	0.826**	0.001	−0.788**	0.001
Jump height (m)			−0.341**	0.001
0.9 m				
	r	*p*-value	r	*p*-value
DJ-index	0.852**	0.001	−0.668**	0.001
Jump height (m)			−0.246*	0.014

* *P* < 0.05.

** *P* < 0.01.

Table 4 presents the results of the multiple regression analysis with the DJ-index for 0.3 m as the dependent variable and lower limb kinetics data as the explanatory variables. The positive power (*β*=0.510, *P* < 0.001) at the ankle joint and positive power (*β*=0.363, *P* < 0.001) at the knee joint were significant factors.

**Table 4:** Multiple regression predictors of the drop jump index at a drop height of 0.3 m.

		Multiple regression analysis		
		*β*	t	*p*-value
1	Ankle positive power (W/kg)	0.510	6.751	0.001
2	Knee positive power (W/kg)	0.363	4.807	0.001

Table 5 presents the results of the multiple regression analysis with the DJ-index for 0.6 m as the dependent variable and lower limb kinetics data as the explanatory variables. The positive power (*β*=0.654, *P* < 0.001), negative work (*β*=−0.280, *P* < 0.001), and negative power (*β*=−0.266, *P* < 0.001) at the ankle joint; positive power (*β*=0.266, *P* < 0.001) at the knee joint; and eccentric torque (*β*=0.223, *P* < 0.001) at the hip joint were significant factors.

**Table 5:** Multiple regression predictors of the drop jump index at a drop height of 0.6 m.

		Multiple regression analysis		
		*β*	t	*p*-value
1	Ankle positive power (W/kg)	0.654	9.320	0.001
2	Knee positive power (W/kg)	0.266	4.664	0.001
3	Hip eccentric torque (Nm/kg)	0.223	4.857	0.001
4	Ankle negative work (J/kg)	0.280	4.321	0.001
5	Ankle negative power (W/kg)	−0.266	−4.058	0.001

Table 6 shows the results of the multiple regression analysis with the DJ-index for 0.9 m as the dependent variable and lower limb kinetics data as the explanatory variables. The positive power (*β* = 0.524, *P* < 0.001), negative work (*β* = 0.482, *P* < 0.001), and negative power (*β* = −0.374, *P* < 0.001) at the ankle joint; positive power (*β* = 0.409, *P* < 0.001) and eccentric trque (*β* = −0.171, *P* < 0.045) at the knee joint; and eccentric torque (*β* = 0.247, *P* < 0.001) at the hip joint were significant factors.

**Table 6:** Multiple regression predictors of the drop jump index at a drop height of 0.9 m.

		Multiple regression analysis		
		*β*	t	*p*-value
1	Ankle positive power (W/kg)	0.524	4.277	0.001
2	Ankle negative work (J/kg)	0.482	4.869	0.001
3	Ankle negative power (W/kg)	−0.374	−3.415	0.001
4	Hip eccentric torque (Nm/kg)	0.247	4.268	0.001
5	Knee positive power (W/kg)	0.409	3.734	0.001
6	Knee eccentric torque (Nm/kg)	−0.171	−2.032	0.045

## 4 Discussion

Multiple regression analysis showed that the positive power of the ankle joint had the most significant effect of the DJ-index at every drop height. In DJ and RJ that require jumping as high as possible in the shortest possible time, the kinetic variables of the ankle joint exert the greatest torque, power, and work during takeoff among those of the three lower limb joints (14–17, 19). Furthermore, it is the most influential parameter among the kinetic variables of the three lower limb joints that affect the RJ-index, jump height, and contact time in RJ (10). Therefore, a high positive ankle joint power suggests a prerequisite for achieving a high DJ-index, irrespective of drop height.

At 0.3 m drop height, a positive power at the knee joint, as well as one at the ankle joint, was found to affect the DJ-index. The ankle and knee joints exert more power during the takeoff phase of DJ at each drop height than the hip joint (16, 17, 19). Additionally, when selecting the optimal drop height for DJ, plyometric training can be performed at a height that does not decrease ankle and knee joint power exertion without compromising the jumping technique (26). Moreover, a previous study showed that the positive power of the ankle and knee joints improved as the DJ-index and jump height increased (18). Therefore, the concentric power exertion of the ankle and knee joints, the main muscle groups, is considered to influence the acquisition of the DJ-index at 0.3 m drop height because the stretch load (kinetic energy) from the fall is small.

At 0.6 m drop height, in addition to the two variables, negative work and power at the ankle joint and eccentric torque at the hip joint were found to affect the DJ-index. Furthermore, at 0.9 m drop height, in addition to these five variables, eccentric torque at the knee joint was found to affect the DJ-index. These results suggest that an eccentric force exertion is required to obtain the DJ-index when the drop height increases. The muscle–tendon complex involved in plantar flexion of the ankle joint is excellent at storing large amounts of elastic energy during the eccentric phase and then reusing that energy during the concentric phase to exert force (39). Moreover, the ankle joint torque at the midpoint of the takeoff affects both jump height and contact time of the RJ (40) because the ankle joint muscle groups can exert force in a short time in its functional anatomy. The muscle-tendon complex involved in plantar flexion of the ankle joint is stretched immediately after the takeoff, which induces the stretch reflex during the eccentric phase, allowing elastic energy to be stored effectively during this phase (28–30). Therefore, in order to achieve a high DJ-index with a drop height that involves a large stretch load, it is necessary to demonstrate eccentric torque and power in the ankle joint, and for this it is necessary to facilitate the stretch reflex with a large stretch load and to reuse the accumulated elastic energy.

In contrast, knee and hip joint force exertion is greater in countermovement type DJ, where the task is to jump as high as possible without considering contact time or jump with a technique that increases contact time, such as heel ground contact (16, 41). However, in these studies, negative power or work of these joints increased in the eccentric phase. Wirth et al. (31) reported that an increase in hip and knee joint motions leads to a longer contact time, suggesting that knee and hip joint motion needs to be minimized to effectively induce the stretch reflex (42). Moreover, it has been shown that a high knee joint stiffness after ground contact plays a key role as a power source in regulating DJ performance, which is acheived by influencing the facilitation of the stretch reflex (43). Thus, the role of eccentric extension torque in the knee and hip joints at high drop heights may be to compensate for the load that cannot be borne by the ankle joint, so that the lower limb joints do not change significantly in the flexion direction when they are subjected to stretch load due to falling from the drop height. Therefore, to achieve a high DJ-index at a 0.6-0.9 m drop height, exerting eccentric knee and hip extension torque is essential to prevent knee and hip flexion when subjected to a large stretch load during ground contact.

Our results showed that kinetic variables of the three lower limb joints affecting the DJ-index are different, depending on the drop height. In addition to the ankle and knee joints, hip joint becomes more important as the drop height increases and eccentric force exertion is required. Therefore, it is possible to evaluate factors that affect the ability to exert power in SSC of the lower limb and provide useful information for plyometric training prescription using the kinetic variables of the three lower limb joints together with the DJ performance variable. However, for some parameters, such as those of the ankle joint, both eccentric and concentric force exertion were important, and for other parameters, such as those of the knee and hip joints, only the eccentric or concentric force exertion was affected in this study. This point should be considered when evaluating the ability to exert power in SSC of the lower limb, thus, our results will be insightful for athletes and coaches. For example, Sub. A (the top athlete who participated in international track and field jump events competitions), who showed the maximum DJ-index at 0.9 m, also had a DJ-index of 2.96 at 0.3 m and 3.30 at 0.6 m. The DJ-index increased as the drop height increased. Looking at the kinetic variables during the takeoff at each drop height for Sub. A, the variables affecting the acquisition of the DJ-index tended to increase at 0.6 m and 0.9 m. Furthermore, Sub. A showed a tendency for knee and hip joint flexion not to change significantly even as the drop height increased (Table 2). Thus, the Sub. A had a superior ability to exert power in SSC under greater stretch load conditions. On the other hand, the Sub. B (badminton athlete) showed a high DJ-index (3.60) at 0.3 m that decreased as the drop height increased. Looking at the kinetic variables during takeoff at each drop height for participant B, positive power of the ankle and knee joints, which affect the acquisition of the DJ-index, showed to tended decrease at 0.9 m for all drop heights. Furthermore, the eccentric torque of the ankle joint also showed to tended decrease at 0.9 m compared to 0.6 m. In addition, Sub. B showed a tendency for knee and hip flexion to increase as the drop height increased (Table 2). Thus, Sub. B may have had superior ability to exert power in SSC under lower stretch load conditions. Therefore, when evaluating the reactive strength of the lower limb in DJ, it may be effective to select the drop height according to the discipline and competition level of the performance variable target and to use multiple drop heights in addition to a single drop height. When performing DJ, if the drop height becomes excessively high, it is suggested that the mechanism related to the power demonstration by SSC, such as the stretch reflex and elastic energy, does not work effectively because the flexion of the lower limb joints at the takeoff becomes larger to absorb the impact (25, 28–30). On the other hand, there are reports that the higher the stretch load, the more the stretch reflex is facilitated (28). Furthermore, previous studies have reported that in athletes with high DJ performance, the state of intracortical inhibition that controls the ankle joint muscles during the pre-set phase becomes a state of disinhibition, and this becomes more pronounced as the height of the takeoff increases (32). It has also been suggested that the state of disinhibition during the pre-set phase affects the facilitation of the stretch reflex during the takeoff phase and the large torque exerted by the ankle joint muscles during the same phase (33). Therefore, the elite athletes with superior power exertion ability in the lower limb SSC may be able to exert greater power even under greater stretch loads by effectively activating the force transmission mechanism via SSC, such as the stretch reflex during takeoff. However, if the drop height used is too high, even athletes who are used to DJ may not be able to withstand the impact of landing, and there is a possibility that the risk of injury will increase due to a lack of safe control of movement (23, 44). Therefore, the drop height used for DJ should be decided by considering the strength of the athlete, their DJ experience and their DJ performance.

This study has a few limitations. Although the DJ-index is calculated using both jump height and contact time, different factors have been shown to improve both parameters (9). Furthermore, a previous study using RJ to examine the kinetic variables of the three lower limb joints that affect the RJ-index has shown that each kinetic variable affecting the jump height and contact time is different. Therefore, future studies are required to clarify the kinetic variables of the three lower limb joints that affect the jump height and contact time at each drop height to enable an appropriate assessment and administration of plyometric training based on the DJ-index. The effects of different kinetic variables during DJ from different drop heights on other motor abilities, such as sprinting and change of direction, should also be examined in the future. In addition, the level of dependence on SSC differs depending on the sport events. For this reason, this content needs to be considered as a future issue.

In summary, although sprinting, change of direction, and jumping ability are highly related to the DJ-index, the results of this study indicate that the influence of kinetics of the three lower limb joints on the acquisition of high DJ-index varies with drop height. As a result, ankle joint and knee joint positive power were extracted as parameters affecting DJ-index at 0.3 m. In addition, negative power and work of the ankle joint and eccentric torque of the hip joint were extracted at 0.6 m, and the eccentric torque of the knee joint was extracted at 0.9 m. Thus, the torque, power, and work required to obtain the DJ-index may vary with the drop height.

## 5 Conclusions

The present study clarified the lower limb joint kinetic variables that affect the DJ-index depending on the drop height, with some parameters affecting any height and other parameters dependent on the drop height. Specifically, when the drop height is increased, certain kinetic variables are affected, such as the ankle and knee joint extension power, ankle joint negative mechanics, knee and hip joint eccentric extension torque. Therefore, we propose that it is important to select multiple drop heights and to consider the characteristics of the kinetic variables of the three lower limb joints at each drop height when evaluating the ability to exert power in lower limb SSC using the DJ.

## Data Availability

The original contributions presented in the study are included in the article/Supplementary Material, further inquiries can be directed to the corresponding author.
